# Effects of Environmental Factors and Metallic Electrodes on AC Electrical Conduction Through DNA Molecule

**DOI:** 10.1186/s11671-017-2076-y

**Published:** 2017-04-27

**Authors:** S. Abdalla, A. Obaid, F. M. Al-Marzouki

**Affiliations:** 1 Department of Physics, Faculty of Science, King Abdulaziz University Jeddah, P.O. Box 80203, Jeddah, 21589 Saudi Arabia; 2 Department of Chemistry, Faculty of Science, King Abdulaziz University Jeddah, P.O. Box 80203, Jeddah, 21589 Saudi Arabia

**Keywords:** AC-complex conductivity, DNA molecule, Environmental and surrounding charges, Localized charges, Potential hills/wells, Dielectric constant

## Abstract

**Background:**

Deoxyribonucleic acid (DNA) is one of the best candidate materials for various device applications such as in electrodes for rechargeable batteries, biosensors, molecular electronics, medical- and biomedical-applications etc. Hence, it is worthwhile to examine the mechanism of charge transport in the DNA molecule, however, still a question without a clear answer is DNA a molecular conducting material (wire), semiconductor, or insulator? The answer, after the published data, is still ambiguous without any confirmed and clear scientific answer. DNA is found to be always surrounded with different electric charges, ions, and dipoles. These surrounding charges and electric barrier(s) due to metallic electrodes (as environmental factors (EFs)) play a substantial role when measuring the electrical conductivity through λ-double helix (DNA) molecule suspended between metallic electrodes. We found that strong frequency dependence of AC-complex conductivity comes from the electrical conduction of EFs. This leads to superimposing serious incorrect experimental data to measured ones.

**Methods:**

At 1 MHz, we carried out a first control experiment on electrical conductivity with and without the presence of DNA molecule. If there are possible electrical conduction due to stray ions and contribution of substrate, we will detected them. This control experiment revealed that there is an important role played by the environmental-charges around DNA molecule and any experiment should consider this role.

**Results and discussion:**

We have succeeded to measure both electrical conductivity due to EFs (*σ*_ENV_) and electrical conductivity due to DNA molecule (*σ*_DNA_) independently by carrying the measurements at different DNA-lengths and subtracting the data. We carried out measurements as a function of frequency (*f*) and temperature (*T*) in the ranges 0.1 Hz < *f* < 1 MHz and 288 K < *T* < 343 K. The measured conductivity (*σ*_MES_) portrays a metal-like behavior at high frequencies near 1 MHz. However, we found that *σ*_DNA_ was far from this behavior because the conduction due to EFs superimposes *σ*_DNA_, in particular at low frequencies. By measuring the electrical conductivity at different lengths: 40, 60, 80, and 100 nm, we have succeeded not only to separate the electrical conduction of the DNA molecule from all EFs effects that surround the molecule, but also to present accurate values of *σ*_DNA_ and the dielectric constant of the molecule *ε*’_DNA_ as a function of temperature and frequency. Furthermore, in order to explain these data, we present a model describing the electrical conduction through DNA molecule: DNA is a classical semiconductor with charges, dipoles and ions that result in creation of localized energy-states (LESs) in the extended bands and in the energy gap of the DNA molecule.

**Conclusions:**

This model explains clearly the mechanism of charge transfer mechanism in the DNA, and it sheds light on why the charge transfer through the DNA can lead to insulating, semiconducting, or metallic behavior on the same time. The model considers charges on DNA, in the extended bands, either could be free to move under electric field or localized in potential wells/hills. Localization of charges in DNA is an intrinsic structural-property of this solitaire molecule. At all temperatures, the expected increase in thermal-induced charge is attributed to the delocalization of holes (or/and electrons) in potential hills (or/and potential wells) which accurately accounts for the total electric and dielectric behavior through DNA molecule. We succeeded to fit the experimental data to the proposed model with reasonable magnitudes of potential hills/wells that are in the energy range from 0.068 eV.

## Background

After the famous discovery of Watson–Crick double helix structure of deoxyribonucleic acid (DNA) molecule, enormous published studies are in clear contradictory: for electrical conduction, DNA has metallic nature [1, 2], it has semiconducting nature [3, 4] or insulating properties [5]. Even Kasumov et al. [6] reported that DNA is a superconductor at sufficiently low temperatures. It is one enigma that is still unknown and without a clear answer till now. In fact, the majority of these studies have been carried out at DC conditions. DC-conditions are not adequate, at all, to explain the exact and the real mechanism of charge transfer (CT) through very complex-structured molecule such as DNA. DC-electric field is only one point in the wide range of AC electrical conduction that varies from few micro Hz’s up to more than giga Hz’s does. Consequently, AC-measurements will have more information than DC-measurements do. Moreover, at DC-conditions, charges cannot carry the electric current if they are blocked against potential wells which are, inevitably, present inside molecule due to several factors as we will see later. In addition, there is no one published study that deals with measuring electrical conductivity due to DNA molecule (*σ*_DNA_) below 1 Hz. However, relatively slim part concerns AC-measurements [7–10] are published. In reference [7], the author has started measurements of *σ*_DNA_ at 1 Hz, but the experimental data were not adequate to explain the experimental data. 1 Hz was too high to detect the relaxation of bound charges at the interface between the metallic electrode and DNA. So, as we will see later, it is imperative carrying out measurements at some 0.1 Hz’s. In addition, some theoretical studies have revealed that transfer of electric charges in the DNA, with 1D disordered, occurs principally between localized states [8–10]. This narrow experimental data for important subject shows how it is important to have clear, correct, and very fine experimental work characterize CT through DNA. As we have stated, there are strong contradictions in the published data concerning CT through DNA. These strong contradictions are found due to some reasons including experimental conditions and uncoupled effects between experimental and conceptual factors. Nevertheless, an extensive quantity of experimental and theoretical studies to understand CT mechanism(s) in the DNA was reported. The most important of these mechanisms are 1D quantum tunneling [11], quantum tunneling [12], and charge hopping [13–15] is a current and clear trend. However, these mechanisms did not explain the effects of some behaviors such as the temperature-dependence and frequency on *σ*_DNA_. From here, we considered that there are several types of charges (we denote them as environmental factors) around DNA molecule that affect the electrical conduction through the molecule [7]. Moreover, some studies using hopping as possible mechanism face different complications when analyzing the behavior of electrical conductivity at low (weak relation) and high temperatures (strong relation) [16]. On the other hand, the published studies on tunneling mechanism and its dependence on temperature are scattered and imperfect [17, 18]. Therefore, we believe that there are real needs for some key issues, namely, (a) studying the role of frequency in AC-conductivity through DNA molecule taking into account the presence of surrounded charges, (b) investigating how temperature, AC-electric field, and applied frequency, vary the CT through DNA. In a previous study, we have demonstrated [7] that CT through DNA molecule is highly affected by localized holes in potential hills that are inevitably present in valence band [7]. For these reasons, we present a model in order to explain the CT through DNA molecule taken into account: (i) the presence of additional charges, dipoles, and ions around the molecule in the EFs, (ii) the temperature and frequency dependence of complex electrical conductivity (iii), and DC-electric behavior.

### Model

Generally, one can find, in the literature, three main theories that explain the CT through DNA: (i) short-range hole/electron tunneling from an (a) acceptor/donor to the corresponding donor/acceptor through DNA, (ii) long-range charge hopping between discrete energy states in the DNA itself, and (iii) some models combine (i) (ii) together. However, applying these mechanisms in DNA case is strictly limited because of the unique structure/nature of this complex molecule. For example, one cannot consider one-dimensional conduction because of the quantity of charges and ions that surround the molecule (in particular water ions [19]). The term water ions means dipoles composed of hydrogen ion and hydroxyl ion attracted to DNA by Van der Waals weak bonds. These models did not account for not only the very complex structure but also the permanent mechanical vibrations with DNA [20]. Here, we summarize the main points of the model:

(1) DNA is considered as a special case of wide band semiconductor with energy band that lies between the conduction band CB “lowest unoccupied molecular orbital (LUMO)” and the valence band VB “highest occupied molecular orbital (HOMO)”, ranges from 4.67 to 4.98 eV as obtained by optical transitions [21]

(2) The presence of inevitable disorder factors is an intrinsic property of DNA molecule, and we cannot ignore their effects. We will state only ten possible sources that create these disorder factors:

2-a Disorder factors due to the presence of electric potential barriers due to metallic electrodes:

2-a (i) The effects of charges, dipoles, and ions at the interface between DNA molecule and metallic electrodes. We will see that this factor will lead to the creation of one trapping hole-level (HL) above the VB (in our sample); we denote this level H1.

2-b Disorder factors due to the presence of electric charges, ions, and dipoles around DNA:

2-b (ii) The effects of surrounding charges, dipoles, and ions (in particular water ions) (H2 and H3).

We will see that this factor will lead to the creation of two trapping hole-levels above the VB (in our sample); we denote this level H2 and H3.

2-b (iii) The presence of permanent hydration shell encountering the molecule [22]. We will see that this factor will lead to the creation of H2 and H3.

2-b (IV) The presence of some impurities in ions (H2 and H3)

2-c Disorder factors due to the presence of electric charges, ions, and dipoles around DNA:

2-c (v) The unique structure of DNA including permanent mechanical vibrations [20]. We will see that this factor will lead to the creation of two trapping hole-levels above the VB in our sample; we denote them H4 and H5.

2-c (vi) Effects of external factors such as temperature, applied electric frequency, environmental, and surrounded charges (not water counter ions—see (ii)); for example, heating extends the molecule while cooling shrinks the DNA (H4 and H5).

2-c (vii) The presence of inevitable disorder that is present due to the inevitable random base pair sequence, which will result in inevitable localized electronic states (H4 and H5).

2-c (viii) The possible mismatch at GC base pair [23] (H4 and H5).

2-d Disorder factors due to general factors DNA

(ix) Electrical conduction occurs in three dimensions (3D) and not 1D, H1, H2, H3, H4, and H5.

**Fig. 1:**
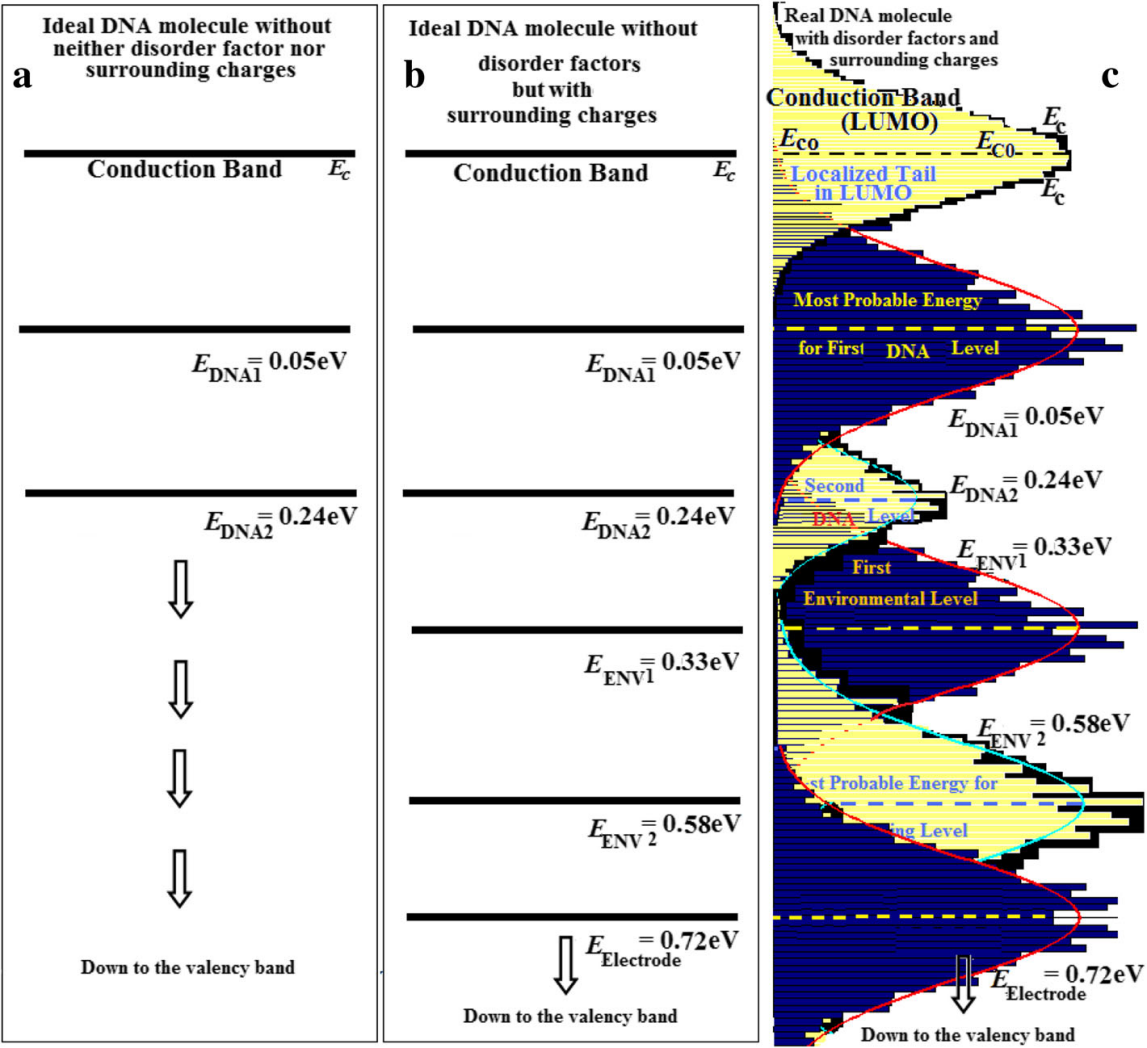
**a** Ideal DNA sample without disorder factors and without surrounding charges. **b** Illustrates an ideal n-type semiconductor DNA sample without disorder factors but it has surrounding charges. **c** represents a real n-type semiconductor DNA sample with both disorder factors and surrounding charges. They are five trapping levels lying above the valence band. One will denote them H1, H2, H3, H4, and H5. Note that this figure represents ideal n-type semiconductor, and the same assumptions are valid for p-type semiconductor

(x) Localized energy-states (LESs) are present in the extended bands, which lead to the creation of potential wells in the conduction band (CB) and potential hills in the valence band (VB).

(3-a) Spontaneous tautomeric transformations of the Watson–Crick DNA base pairs into the pairs with the wobble architecture [24–28]

(3-b) The presence in the structure of DNA molecule the purine-pyrimidine: it is possible to have different metabolic pathways to synthesize and break down purines [29–33].

(3-c) The localized energy states (LESs) are distributed in 3D space in a Gaussian distribution around some most probable values (*E*_C0_ in the CB and *E*_V0_ in the VB). This means that the extended bands (CB and VB) contain several energy states: states above *E*_C0_ contains free electrons (states below *E*_V0_ contains free holes); and states below *E*_C0_ contains localized electrons (states above *E*_V0_ contains localized holes). Figure 1a–c illustrates the energetic situation in an ideal n-type semiconductor DNA. Figure 1a shows ideal DNA sample without disorder factors and without surrounding charges. Figure 1b illustrates an ideal n-type semiconductor DNA sample without disorder factors, but it has surrounding charges. Figure 1c represents a real n-type semiconductor DNA sample with both disorder factors and surrounding charges.

(4) According to the abovementioned scenario, the LESs having energies *E*_C_ and *E*_V_ in the extended bands, conduction band (CB), and valence band (VB) will fluctuate around a most probable value *E*_C0_ in the conduction band and *E*_V0_ in the valence band. Thus, the LESs in the CB and VB have localized energies, which are energetically activated as *γ* = (*E*_C_−*E*_C0_).

(5) The depth of the potential well gγ (where *g* is the Gaussian-integral limits) depends on both the Gaussian distribution of these localized states and the nature of disorder. “*g*” varies in the range −3 < *g* < 3, and it covers all possible energies in conduction and valence bands. Fine-fitting process of experimental data to model leads to obtain the localization (disorder) energy, *γ*. The physical meaning of *g* is related to the distribution of relaxation times; for example, in our case if *g* = 0 this means that the Gaussian distribution will tend to have unique value corresponding to 1D conduction with only one definite relaxation time and no localization of charges. Thus, one can use the limits of gas-fitting parameters, which reflects the extent of distribution of relaxation times.

(6) The Gaussian probability to find certain localized energy state within the extended bands P(E) is related with the disorder energy of potential hills (for electrons at bottoms of wells) and thermal energy as

1 $$ {P}_{\mathrm{el}}\left({E}_{\mathrm{C}}\right)={\displaystyle \underset{-{E}_{\mathrm{C}}}{\overset{E_{\mathrm{C}}}{\int }}\frac{1}{\gamma \sqrt{2\pi}} \exp \frac{-{\left({E}_{\mathrm{C}}-{E}_{\mathrm{C}0}\right)}^2}{2{\gamma}^2}} d{E}_{\mathrm{C}} $$

and similar for holes localized at summits of potential hills as:

2 $$ {P}_{\mathrm{ho}}(E)={\displaystyle \underset{E_{\mathrm{V}}}{\overset{-{E}_{\mathrm{V}}}{\int }}\frac{1}{\gamma \sqrt{2\pi}} \exp \frac{-{\left({E}_{\mathrm{V}0}-{E}_{\mathrm{V}}\right)}^2}{2{\gamma}^2}}\mathrm{d}{\mathrm{E}}_{\mathrm{V}} $$

Thus, structural changes in DNA enhance such localization by different parameters such as temperature. This will lead, in turn, to increase the electrical conductivity with temperature. One can normalize the fluctuations of energy bands by using the reduced energy factor *u* ^2^ = (*E*_C_−*E*_C0_) ^2^/2γ^2^ = (*E*_V0_−*E*_V_) ^2^/2γ^2^ and the integral limits varies as: −3 < *g* < 3 or the Gaussian probability will be:

3 $$ P(u)=\frac{1}{\sqrt{2\pi}}{\displaystyle \underset{- g}{\overset{+ g}{\int }} \exp -\left({u}^2/2\right)}\mathrm{d}\mathrm{u} $$

**Fig. 2:**
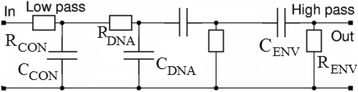
Equivalent electronic circuits of DNA molecule at different frequency ranges

(7) The abovementioned eight points are adequate to explain the electrical conduction through disordered macromolecule but not to DNA. As a particular case, DNA molecule always surrounds at ambient and normal conditions, with different ions; the most important of them are water ions. These inevitable external ions affect drastically the CT through DNA, and one should account to the factors of ambient conduction before calculating the electrical conductivity. This can be resolved by measuring complex conductivity at different lengths of DNA, then subtracting to get the electrical conductivity of the molecule without the effect of surrounding ions and/or charges, as we will see later. This is also confirmed by measuring the electrical conductivity at different temperatures in the range (resaving temperature 288 K < T < near melting temperature 343 K).

(8) We had better present the above points in electronical presentation as the following: we consider the DNA molecule as a parallel *R*_DNA_ *C*_DNA_ circuit. This circuit connects in parallel with another parallel *R*_EVN_ *C*_ENV_ circuit. These two combinations connect in series with a third parallel *R*_CON_ *C*_CON_ circuit. *R*_DNA_ *C*_DNA_ circuit and *R*_EVN_ *C*_ENV_ circuit present the effect of localization of charges in the potential wells (holes in potential hills), while *R*_CON_ *C*_CON_ circuit stands essentially for the effect of the eclectic potential barrier at the level of DNA/metallic contacts. Figure 2 illustrates schematically this scenario. The effect of the third circuit is more important only at low frequencies and DC-conditions while one could ignore its action at high frequencies. On the contrary, the effect of the parallel *R*_DNA_ *C*_DNA_ circuit and *R*_EVN_ *C*_ENV_ circuits is more pronounced at high frequencies and can have minor effect at low frequencies and DC-conditions.

At low frequencies and DC-conditions, one can ignore the effect of the parallel *R*_DNA_ *C*_DNA_ circuit and *R*_EVN_ *C*_ENV_ circuits as they have minor effect.

(9) One can use similar explanation to explain the effect of external electric AC-field with sufficiently high frequency (more than 1 MHz) where the localized charges will conduct the electrical current regardless their localization. On the contrary, at low frequencies and electric DC-conditions, the walls of potential barriers localized will stop charges against their walls. Thus, they cannot carry the electric current and cannot conduct the energy.

As the molecule length increases, the disorder increases, and the disorder energy increases leading to more localization of charge carriers. We will take a simple linear dependence between the length of molecule (*L*) and the energy of disorder *γ* as:

4 $$ L={L}_0\left(1+ a\gamma \right) $$

Where *α* is a suitable constant of dimensions nm.eV^−1^ and *L*_0_ is a critical length of DNA molecule, which equals to an equivalent DNA molecule without any disorder effect and lies in a straight line and L is the real molecule length.

(11) The localization of charges attains its maximum value at DC-conditions and decreases with the applied frequency until it finally tends to zero at infinite frequencies. Here, at: as *f* → ∞, uγ → 0 the localization becomes zero. Similarly, the localization of charges attains its maximum value at very low temperatures *T* → 0, and decreases with heating until it finally tends to its maximum value when *T* → ∞. Here, one can write:

**Table:** 

	Frequency	Temperature	Height of barrier	Electrical conductivity
Minimum localization	*f* → ∞	*T* → ∞	*u*γ → 0 eV	Maximum
Maximum localization	*f* → 0	*T* → ∞	*u*γ → gγ *g* is the integral factor	Minimum

### Application of the Present Model to Explain DC-IV (*T*) Characteristics

We consider a thermionic emission happens at relatively high-applied DC-electric fields, *E*_DC_. In addition, we consider the presence of potential barrier, *ϕ* eV, that exist in both electrodes after fitting the experimental data with the thermionic emission [34]; for simplicity, we will consider similar barrier in both electrodes. After the presented model, the potential wells (hills) will shrink if the disorder energy is inferior to the applied electric potential *E*_DC_: *E*_DC_ > > *uγ*. In addition, the reduction of *u*γ will increase the possibility to release the localized electrons (holes) inside the wells (hills) to be free and, thus, they can carry out the electric current. This increases the current with DC-electric field. Moreover, the generation of electron/hole pairs across the forbidden gap *E*g constitute an essential part of the saturation current *i*_gen_ which is proportional to: 2*[(m*) kT/(2π*h* ^2^)] ^3/2^exp (−*E*_*g*_/2k*T*) while the other part is due to the diffusion of carrier’s *i*_diff_ which is proportional to exp (−*Eg*/k*T*). The effect of localization on the generation rate is to increase the generation rate because the bottoms of potential wells in the conduction band will be nearer to the heights of potential hills in the valence band by an average factor equals to the localization energy, *uγ*. On the contrary, the effect of localization on *i*_diff_ is to decrease the measured current because scattering against potential barriers through the extended bands will reduce the mobility of charges. We consider that the reduction of mobility has an exponential form which is related to the localization energy, *uγ*, and the mobility as following: $\mu ={\mu}_0{\displaystyle \underset{- g}{\overset{g}{\int }}\left(\frac{L_0}{L\left(1+\alpha \gamma \right)}\right)} \exp \left(\frac{ u\gamma}{\mathrm{kT}}-\frac{u^2}{2}\right)\mathrm{d}\mathrm{u}.$

After the present model, one can write the saturation current as:

5 $$ {i}_{\mathrm{s}}={i}_{\mathrm{gen}}+{i}_{\mathrm{diff}}={\displaystyle \underset{- g}{\overset{g}{\int }}\left(\frac{L_0}{L\left(1+\alpha \gamma \right)}\right)\left\{{A}_1 \exp \frac{Eg- u\gamma}{2\mathrm{kT}}+{A}_2 \exp \frac{Eg+ u\gamma}{\mathrm{kT}}\right\}} \exp \left(-\frac{u^2}{2}\right)\mathrm{d}\mathrm{u} $$

Thus, the measured current can describe this scenario by the equation:

6 $$ i\left(+\mathrm{ve}\right)={\displaystyle \underset{- g}{\overset{g}{\int }}\left(\frac{L_0}{L\left(1+\alpha \gamma \right)}\right)\left\{{A}_1 \exp \frac{Eg- u\gamma}{2\mathrm{kT}}+{A}_{2+} \exp \frac{Eg+ u\gamma}{\mathrm{kT}}\right\}\left( \exp \frac{qV- u\gamma (V)}{\mathrm{kT}}-1\right)} \exp \left(-\frac{u^2}{2}\right)\mathrm{d}\mathrm{u} $$

7 $$ i\left(-\mathrm{ve}\right)={\displaystyle \underset{- g}{\overset{g}{\int }}\left(\frac{L_0}{L\left(1+\alpha \gamma \right)}\right)\left\{{A}_1 \exp \frac{Eg- u\gamma}{2\mathrm{kT}}+{A}_{2-} \exp \frac{Eg+ u\gamma}{\mathrm{kT}}\right\}\left(1- \exp \frac{ u\gamma -(qV)}{\mathrm{kT}}\right)} \exp \left(-\frac{u^2}{2}\right)\mathrm{d}\mathrm{u} $$

The DC electrical conductivity *σ*_DC_ is obtained as follows:

*σ* = (*i*/*v*)*(*L*/*A*_Eff_) (6-a) where *A*_Eff_ is the effective area that connects the DNA molecule and the metallic electrode, and *L* is DNA-length.

*A*_1_ is the generation current produced at infinite temperatures *T* → ∞; *A*_2+_ and *A*_2−_ are the drift currents when *T* → ∞.

### Application of the Present Model to Explain AC−*σ* (*L*, *T*−ω) Characteristics

The significance of using AC-field, *E*_AC*,*_ lies in the frequency-oscillations effect and not in its magnitude. Thus, we will neglect the effect of lowering potential barrier height by AC-electric field compared to the effect of applying frequency on the localized charges, as we will see later. *E*_AC_ give the localized electrons more chance to carry the electric power (the current) as follows: as the AC-field is applied, the localized charges will respond with certain relaxation time *τ*. Then, similar to a bypass condenser they will follow the external frequency and will conduct the electric current depending on the applied frequency. After the present model, we will apply the Debye relaxation mechanism on localized electrons in the potential wells.

Consequently, AC-electrical conductivity increases with frequency. Similarly, at certain frequency, AC-electrical conductivity increases with temperature because this latter reduces the depth of potential wells that permits localized electrons to become free. These facts can be translated to mathematical equations as follows: one can write an expression that can describe the relaxation time of a localized electron (hole) in potential well (hill) as follows:$\tau ={\tau}_0 \exp \left(\frac{u\gamma}{kT}\right)$ where *uγ* is the localization energy. Here, *τ*_0_ is the relaxation time of charge without disorder (without localization), and *T* is the temperature. “*τ*_0_” is the relaxation time of carriers at infinite frequencies where there is no localization. We can express the average relaxation time as follows:

8 $$ {\tau}_{\mathrm{average}}=\frac{1}{\sqrt{2\pi}}{\displaystyle \underset{- g}{\overset{g}{\int }}{\tau}_0} \exp \left(-\frac{u^2}{2}\right)\mathrm{d}\mathrm{u} $$

One can calculate the relaxation time from the following relation without considering the localization of charges as:

9 $$ \tau ={\tau}_0 \exp \left(\frac{\varDelta E}{kT}\right) $$

where Δ*E* is the activation energy of the charges, the effect of charge-localization in potential hills is to increase *τ*, therefore, we can write an expression to estimate the relaxation time taken into account the localization effect as:

10 $$ {\tau}_{\mathrm{average}}=\frac{1}{\sqrt{2\pi}}{\displaystyle \underset{- g}{\overset{g}{\int }}{\tau}_0} \exp \left(-\frac{\varDelta E+ u}{kT}\right) \exp \left(-\frac{u^2}{2}\right)\mathrm{d}\mathrm{u} $$

Taken into account the parallel *RC*-circuits previously stated in point 11; if we consider that *C*_dc_ is the measured capacitance at very low frequencies and *C*_∞_ is the measured capacitance at very high frequencies, one can write an expression describing the measured capacitance (*C*_mes_) and the measured electrical conductance (*G*_mes_) for only one trapping level as follows:

11 $$ {\varepsilon}_{\mathrm{mes}}=\frac{1}{\sqrt{2\pi}}{\displaystyle \underset{- g}{\overset{g}{\int }}\left(\frac{L_0}{L\left(1+\alpha \gamma \right)}\right)\left({\varepsilon}_{\infty }+\frac{\left({\varepsilon}_{dc}-{\varepsilon}_{\infty}\right)\mathrm{du}}{1+{\left(\omega {\tau}_0\right)}^2 \exp \left(\frac{2 u\gamma}{\mathrm{kT}}\right)}\right)} \exp \left(-\frac{u^2}{2}\right)\mathrm{d}\mathrm{u} $$

12 $$ {\sigma}_{\mathrm{mes}}=\frac{1}{\sqrt{2\pi}}{\displaystyle \underset{- g}{\overset{g}{\int }}\left(\frac{L_0}{L\left(1+\alpha \gamma \right)}\right)\left({\sigma}_{\mathrm{dc}}+{\omega}^2{\tau}_0 \exp \left(\frac{ u\gamma}{\mathrm{kT}}\right)\frac{\left({\varepsilon}_{\mathrm{dc}}-{\varepsilon}_{\infty}\right)\mathrm{du}}{1+{\left(\omega {\tau}_0\right)}^2 \exp \left(\frac{2 u\gamma}{\mathrm{kT}}\right)}\right)} \exp \left(-\frac{u^2}{2}\right) d u $$

From Eqs. (11) and (12), both measured electrical conductance *G*_mes_ and measured capacitance *C*_mes_ decreases with increasing disorder and it, also, decreases with DNA length. The electrical conductivity *σ* is obtained from the electrical conductance *G* via the well-known relation *σ* = G*Length (*L*)/effective area (*A*_Eff_).

One can write an expression for the electric losses ε” as:

13 $$ \varepsilon {"}_{\mathrm{mes}}=\frac{1}{\sqrt{2\pi}}{\displaystyle \underset{- g}{\overset{g}{\int }}\left(\frac{L_0}{L\left(1+\alpha \gamma \right)}\right)\left(\omega {\tau}_0 \exp \left(\frac{ u\gamma}{\mathrm{kT}}\right)\frac{\left({\varepsilon}_{dc}-{\varepsilon}_{\infty}\right)\mathrm{du}}{1+{\left(\omega {\tau}_0\right)}^2 \exp \left(\frac{2 u\gamma}{\mathrm{kT}}\right)}\right)} \exp \left(-\frac{u^2}{2}\right)\mathrm{d}\mathrm{u} $$

This model results in some remarks:

(i) One can define a particular temperature *T*_p_ at which thermal energy k*T*_p_ equals the high (/depth) of potential hill for a localized hole (potential well for a localized electron) where the probability *P* (*E*) reaches its maximum. At this temperature, electrical conductivity due to certain trapping level (n or m or both) reaches, also, its maximum while dielectric constant should attain a minimum. Here, one can consider 1-D electrical conduction, due to that trapping level.
(ii) Localization of charges terminates at infinite frequencies. At any of these conditions, the charges will relax with a relaxation time *τ*_limit_. Therefore, the electrical conductivity will attain a limit value at very high frequencies:
${\sigma}_{\infty }={\sigma}_{\mathrm{dc}}+\frac{\left({\varepsilon}_{\mathrm{dc}}-{\varepsilon}_{\infty}\right)}{\tau_{\mathrm{limit}}}$ (13-a) where *ε*_∞_ and *ε*_dc_ is the dielectric constant at very high and very low frequencies.
(iii) The abovementioned model can fit variations of electrical conductivity (and dielectric constant) as a faction of both temperature and frequency on the same time.

## Experimental

### Sample Holder

**Fig. 3:**
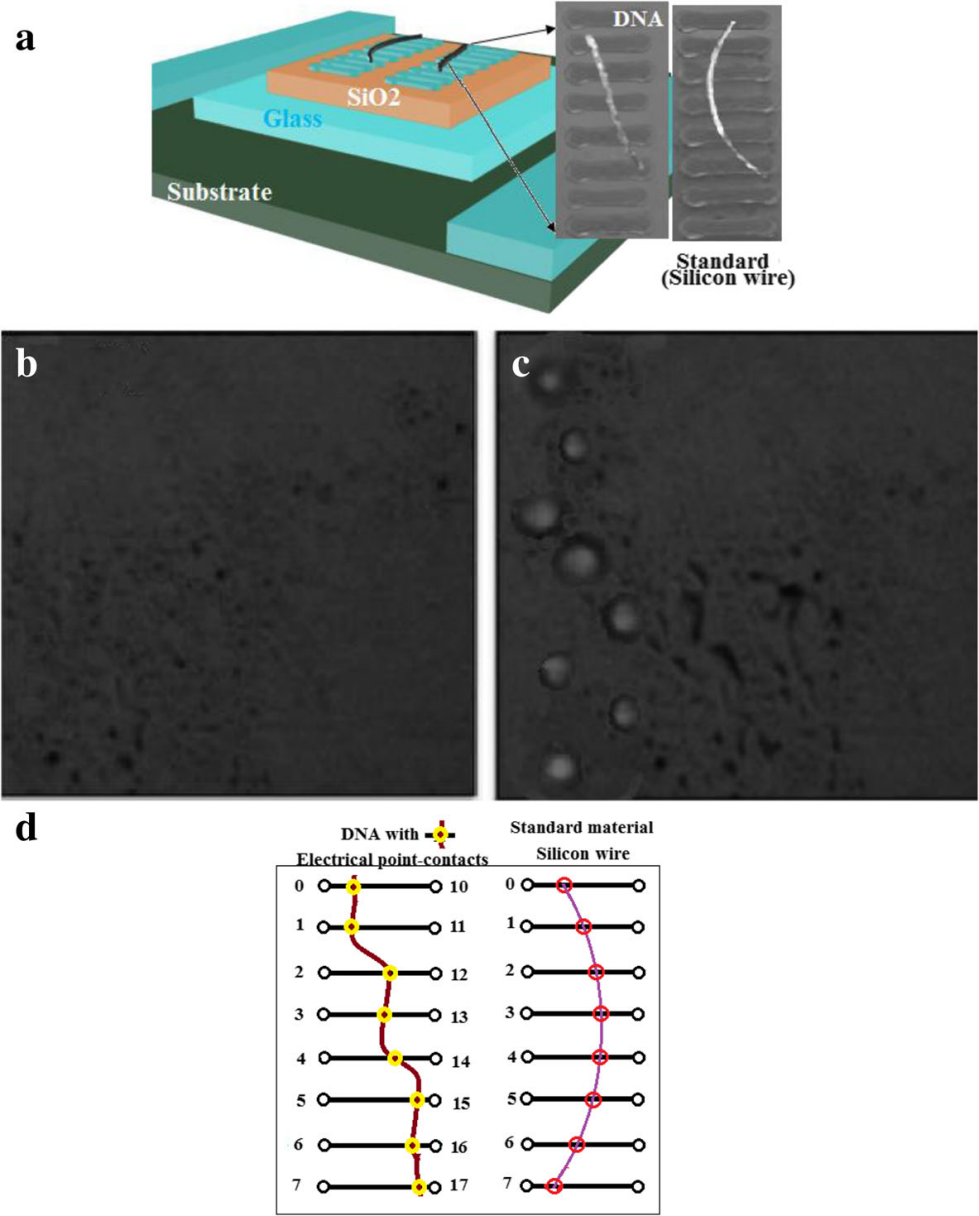
**a** Two sets of eight Au contacts with interspacing 20 nm are shown. DNA molecule is placed on one set of these electrical Au-contacts. The other set of Au-contacts connect a standard material. **b** shows image of fluorescent microscope due to point-contacts before pipetting Epicenter-solution. **c** Shows the same image after pipetting that solution. The thiol contact points on DNA serve to main jobs: (*1*) they carry out good metallic-DNA contacts and (*2*) they fix the molecule through eight fixing points which enable constant effective surface area between the metal and DNA **d** for *I*-*V*-*L* measurements at *T* = 298 K, we put DC-potential polarization between the points 10, 15, and we get the corresponding DC-currents from points: 0 and 2, 0 and 3, 0 and 4, and 0 and 5 for lengths 40, 60, 80, and 100 nm, respectively (Fig. 3c). While for *I*-*V*-*T* measurements at *L* = 60 nm, we put DC-potential polarization between the points 10 and 15, and we get the corresponding DC-current from points: 0 and 5. For AC-measurements at constant temperature (at *T* = 298 K), we put AC-leads of Solartron between the points: 0 and 2, 0 and 3, 0 and 4, and 0 and 5 for lengths 40, 60, 80, and 100 nm, respectively. While for AC-measurements at constant length (at *L* = 60 nm), we put AC-leads of Solartron between the points: 0 and 3 for all temperatures. We repeat these same experimental details on a standard material to have some dimension information about the effective surface area between the metal and standard sample which gives the effective surface area between the metal and DNA. The thiol contact points on DNA serve to main jobs: (*1*) they carry out good metallic-DNA contacts and (*2*) they fix the molecule through eight fixing points which enable constant effective surface area between the metal and DNA

Following Vahidi et al. [35], we carried out suitable bionano electronics at several steps: using photolithography, we begin, first, with the manufacturing of microelectrodes: tailoring two sets micro-electrodes, each set has eight gold contacts with interspacing 20 nm (Fig. 3a, d). Single-stranded DNA is placed on one set of these electrical Au-contacts. The other set of similar Au-contacts are connected to a standard material. Then, second, we attached DNA molecule to one set of electrodes while we specially designed the other electrode to measure all over the DNA length. Figure 3a illustrates graphical microstructure schema used in this study.

We purchased custom synthesized-DNA (de-salted) from Ultramer® (DNA Plate Oligo-IDT, Genomics BioSci & Tech Co., Ltd. New Taipei, Taiwan). Ultramer® delivered samples with different concentrations from 1 up to 10 μmol. We initially choose the structure oligonucleotides (a) 5′-GGG CGG CGA CCT/3 ThiolMC3-D/-3′ in order that it contains thiol group (as carbon-bonded –C–S–H(R)-group) on its 3′end. This will form a robust covalent bond with gold electrodes. In micro tube, it was diluted to 1 μM solutions with Trizma buffer solution and was washed with Tris-EDTA buffer solution (pH 8). Then, we kept the temperature at 104 °C outside the micro tube. We allowed the tube to cool down to 27 °C by removing it from the waterbath. This technique allows us to form sufficiently long single-stranded oligonucleotides with one 3′ thiol-end. We keep it at constant temperature 4 °C. Vahidi et al. [35] reported that Au form a robust electronic bond with free thiols. In addition, 1:1 ratio in volume mixture of annealed strand and Tris (2-carboxyethyl) phosphine hydrochloride (TCEP) are mixed together which stimulates the disulfide linkage to be reduced to a (−SH) [36]. We summarize the technique in the following steps:

1. Vortex carefully 10 μL of TCEP then keep it for 5 min.
2. The supernatant will be on the top leaving the slurry gel.
3. Gather neatly the supernatant material followed by adding 10 μL of strand mixture to the slurry.
4. Mix it well and leave it at 27 °C for 10 min to complete the reduction of all thiol groups.
5. An important note is that free thiol is chemically active substance, and one should use the product immediately after taking the supernatant of this mixture that contains the reduced strands.
6. Thus, the obtained single strand has its 5′ end on each strand overhanging its 3′ strand by 12 bases (sticky ends).
7. We used end-repair kit obtained from Epicenter [37] in order to add the complementary bases on the 3′ ends one blunts the 5′- ends.
8. The obtained molecule was mixed with 3 μl T4 DNA Ligase Buffer, 1 μl of 10 mM dNTPs and 0.5 μl of T4 Polymerase, and 0.5 μl of T4 PNK in a 2 ml microfuge tube.
9. At 27 °C, we incubated the blend for 50 min, and the process ends by heating for 10 min the mixture to about 70 °C. The 3′ ends are now clean, and it is ready to use. One can control the quantity of mixture as desired. In regular steps, we then diluted DNA to have a terminal concentration of 0.25 ng/μl.

In order to get a robust attachment between DNA and gold electrodes, we get rid of potential bubble that would form between molecule and gold surface by plasma etching the surface at 75 W for 7 s before dropping the molecule. Once we place the molecule in its correct position while viewing under an optical microscope to get the correct place. Then, we manually pipetted one drop of DI-water with End-ItTM DNA end-repair [37] solution to get strong covalent bond via thiol attachment [35]. After 10 min at room temperature, the reaction between thiol and gold ends. We moved the molecule, while viewing under an optical microscope, by electrical biasing on the contact points of the electrodes. We polarize the device taken into account that DNA be intrinsically negatively charged. Using deionized water, we carefully washed the chip and let it at room temperature to return dry. All our measurements are carried out in dry condition at ambient pressure without vacuum.

### Initial Quality Test of DNA–Gold Binding

In order to ensure that the thiol group binds well with gold, we carried out two initial quality test experiments:

1. DNA shows an intrinsic fluorescent at 600 nm [38]. Thus, we used a DNA special binding fluorescent stain gel to get the images of point contacts with excitation wavelength in the range 400–530 nm [39]. By using greenish-yellow filters and blue light to detect the emission spectra, we imaged the chip before and after pipetting End-ItTM DNA end-repair/Epicenter solution [37]. Some drops of 5 μl of deionized water with stained gel were carefully peptide on the point contacts and were set for about 10 min to adhesive stain. Under a fluorescent microscope, we control the imaging processes.
2. The second control experiment concerns electrical behavior of the measured conductance between gold electrodes before and after setting the molecule, i.e., we measure the AC-current with DNA and without it to control the presence of DNA and to measure the effect of high-frequency stray capacitances if any. Figure 3b, c shows the fluorescent due to point contacts before and after pipetting Epicenter solution. It seems that spacing between gold electrodes is not regular, but this is not the case because the fluorescent has irregular nature due to its dependence on volume of droplet of End-ItTM DNA end-repair/Epicenter solution. From these figures, one can conclude that there are electrical contact points at the location between DNA and gold.
  For *I*-*V*-*L* measurements at *T* = 298 K, we put DC-potential polarization between the points 10 and 15, and we get the corresponding DC-currents from points 0 and 2, 0 and 3, 0 and 4, and 0 and 5 for lengths 40, 60, 80, and 100 nm, respectively (Fig. 3c). While for *I*-*V*-*T* measurements at *L* = 60 nm, we put DC-potential polarization between the points 10 and 15, and we get the corresponding DC-current from points: 0 and 5. For AC-measurements at constant temperature (at *T* = 298 K), we put AC-leads of Solartron between the point’s points: 0 and 2, 0 and 3, 0 and 4, and 0 and 5 for lengths 40, 60, 80, and 100 nm, respectively. While for AC-measurements at constant length (at *L* = 60 nm), we put AC-leads of Solartron between the points 0 and 3 for all temperatures. We repeat these same experimental details on a standard material to have some dimension information about the effective surface area between the metal and the standard sample which gives the effective surface area between the metal and DNA. The thiol contact points on DNA serves to main jobs: (1) they carry out good metallic-DNA contacts and (2) they fix the molecule through eight fixing points which enable constant effective surface area between the metal and DNA.
3. The expansion coefficient of DNA is 2 Å/°C [40] which makes 0.5% of error percentage. This is an acceptable tolerance of experimental measurements.

Actually, it is one of the biggest obstacles of the DNA conductivity measurements to keep the electrical contact the same from time to time. We need to explain how it can be kept the same?

The abovementioned sample holder illustrated in Fig. 3a–d contains set of gold electrodes with interspace 20 nm (Fig. 3d). We place carefully the single strand on the eight electrodes. Then, we pipetted one drop of DI-water with End-ItTM DNA end-repair [37] solution to get strong covalent bond via thiol attachment with gold. This was done in the eight points, which electrically connect DNA with eight electrodes. By this technique, we clue the strand to the chip and allow to have robust bond connecting the single strand via 3′ thiol end with gold electrode. We can consider that the effective surface between DNA and metal is constant. We keep the strand at constant temperature 4 °C. Vahidi et al. [35] reported that Au forms a robust electronic bond with free thiols. In addition, 1:1 ratio in volume mixture of annealed oligos and Tris (2-carboxyethyl) phosphine hydrochloride (TCEP) are mixed together which stimulates the disulfide linkage to be reduced to a (−SH) [37].

By fixing DNA at eight points, we succeeded to carry out all measurements at the same conditions, and we kept the metallic contact area constant. The separation between electrodes has been carefully chosen in order to fit adequately the length of a certain part of DNA molecule. Suitable banana electronics generates several steps: using photolithography, we begin, first, with the manufacturing of microelectrodes. Then, second, we attached DNA molecule to one electrode while we specially designed the other electrode to measure from four points all over the DNA-length.

Following the method developed by Vahidi et al. [35], typical negative photolithography techniques have been used to fabricate a microchip. Washing a silicon wafer with thin layer of SiO_2_ using acetone followed by isopropyl alcohol and finally with deionized H_2_O; then we have used a stream of nitrogen gun for 2 min in order to dry the substrate at a constant temperature at about 400 K. Then, we cooled the substrate down to ambient temperature. After that, we attached the molecule through two oligonucleotides having different sequences: S-1 5′-GGG CGG CGA CCT/3ThioMC3-D/-3′ and S-2 5′-AGG TCG CCG CCC-3′ (20 nmol Ultramer® DNA Plate Oligo-IDT, Genomics BioSci & Tech Co., Ltd. New Taipei, Taiwan). In particular, we attached both of them to the *μ*-metallic-contacts that are protected with a layer of thiol-gold. We chose one of the strands of the two oligonucleotides and attached it using the self-gathering characteristic property of DNA. The processes contain annealing of oligos, their reduction and attachment to the metallic microelectrodes, followed by one DNA end-repair.

At 1 MHz, we carried out a first control experiment on electrical conductivity with and without the presence of DNA molecule. If there are possible electrical conduction due to stray ions and contribution of substrate, we will detect them. This control experiment revealed that there is an important role played by the environmental charges around DNA molecule, and any experiment should consider this role. An important consideration should be taken into account when proceeding the experiment is to search the optimum concentration *C*_opt_ of DNA which leads to minimum number of ropes; we have found it in the range: 0.06 ng/μL < *C*_opt_ < 0.07 ng/μL. This range has given about three double strands. Drying of DNA molecule is, also, a key factor studied in this work because water ions constitute an important participation to electrical conduction through DNA. Our primary and control experiments show that there is a significant difference between electrical conduction with and without drying. The measurements show that DNA is about one decade more conductor before and after drying. This is because water ions increase the electrical conduction. We have purged the set up with a stream of dry nitrogen at ambient temperature before all measurements.

After ensuring good metallic attachment to DNA, we carefully washed the chip with deionized H_2_O; then drying with nitrogen about 1 h before one carried out electrical characterization in dry ambient. Using Solartron 1260A (AMETEK, TN, USA), we have characterized the electrical conduction through DNA molecule in the potential range —2 V < *v* < 2 V at three mA current. We used two frequency ranges: the first from 0.1 up to 5 Hz, and the second from 1 up to 1 MHz. We kept the rate of scan at 0.1 V/s in performing *I*-*V* (*T*) characteristics with Keithley 2400 (Tektronix SMU Instruments, USA).

One customized set up device containing DNA in special electromagnetic shielded carbon container, and we kept it at reservation temperature 4 °C for 24 h. Then, we carefully dried it with nitrogen stream as stated before. One important point is that temperature would alter intrinsic morphology of DNA; so, we performed all measurements out at a stable temperature. Examination using microscope showed that the gold deposited was carried out only on the desired electrodes.

### Separation Between Electrical Conductivity due to DNA Molecule and Environmental Factors

As we previously mentioned, the measured AC-electrical conductivity is the sum of two principal conductivities: one due to environmental factors (*σ*_ENV_) which includes the conductivity due to metallic-DNA electrodes, and the other is due to DNA molecule itself: *σ*_MEASURED_ = *σ*_ENV_ + *σ*_DNA_. In order to separate these two conductivities, we carried out the experiment on different lengths of DNA molecule. Because *σ*_ENV_ is not affected with the length variation, we performed simple subtraction to get *σ*_DNA_.

We noticed the net presence of three humps (H1, H2 and H3) in the curves describing AC-conductivity as a function of frequency (*f*) and for different temperatures (*T*). These humps move smoothly with both *f* and *T* as we will see later. The first three humps (H1, H2, and H3) are very insensitive to the variation of DNA-length while H4 and H5 vary with *L*_DNA_. This is also true for the variation of dielectric constant as a function of *L*_DNA_, i.e., H1, H2, and H3 are independent to the variation of DNA-length while H4 and H5 strongly depend on *L*_DNA_. From this fact, we considered that H1, H2, and H3 are attributed to the conduction by charges, ions, and dipoles surrounding with the molecule while H4 and H5 are attributed to the electric conduction by charges within the molecule itself. Moreover, this is also true for the experimental data of dielectric constant as a function of frequency for different *L*_DNAs_. From these experimental facts, one can conclude the following:

1. We believe that the hump at very low frequency (about 0.64 Hz), H1, is attributed to bound dipoles and bound charges located at the surface DNA-metallic electrode contacts. The electrical conductivity due to H1 will be denoted *σ*_ELC_, and the charges relax with a relaxation time *τ*_ELC_. When varying *L*_DNA_, H1 will be insensitive to any variations of the molecule length. So, one can eliminate the effect of this hump when subtracting data of different lengths.
2. The measured conductance for certain length (*L*) for example 40 nm is given as the total conductance:
  $$ \begin{array}{l}\begin{array}{l}{G}_{\mathrm{MES}(40)} = {G}_{\mathrm{Electrodes}} + {G}_{\mathrm{ENV}1} + {G}_{\mathrm{ENV}2} + {G}_{\mathrm{DNA}1(40)} + {G}_{\mathrm{DNA}2(40)}\hfill \\ {}{G}_{\mathrm{MES}(60)} = {G}_{\mathrm{Electrodes}} + {G}_{\mathrm{ENV}1} + {G}_{\mathrm{ENV}2} + {G}_{\mathrm{DNA}1(60)} + {G}_{\mathrm{DNA}2(60)}\hfill \\ {}{G}_{\mathrm{MES}(80)} = {G}_{\mathrm{Electrodes}} + {G}_{\mathrm{ENV}1} + {G}_{\mathrm{ENV}2} + {G}_{\mathrm{DNA}1(80)} + {G}_{\mathrm{DNA}2(80)}\hfill \end{array}\\ {}{G}_{\mathrm{MES}(100)} = {G}_{\mathrm{Electrodes}} + {G}_{\mathrm{ENV}1} + {G}_{\mathrm{ENV}2} + {G}_{\mathrm{DNA}1(100)}+{G}_{\mathrm{DNA}2(100)}\end{array} $$
  Simple mathematics will give accurate conductance for DNA molecule.
  We considered that *G*_Electrodes_ lays at the most lower frequency.
3. The incremental value *G*_MES40_ − *G*_MES60_ = *G*_DNA40_ − G_DNA60_ represents the conductance of DNA for an incremental length 20 nm.
4. Therefore, the electrical conductivity of incremental portion of DNA could be written as *G*_DNA40_ − G_DNA60_ = σ_DNA_*(*A*_Eff_/*L*_DNA_).
5. The electrical conductivity of DNA is given as:

14 $$ {\upsigma}_{\mathrm{DNA}} = \left({G}_{\mathrm{DNA}601,\ 2} - {G}_{\mathrm{DNA}401,\ 2}\right){L}_{DNA}\left(20 nm\right)\ /{A}_{\mathrm{Eff}} $$

Similarly, one can get the “dielectric constant” of DNA as:

15 $$ {\upvarepsilon}_{\mathrm{DNA}} = \left({C}_{\mathrm{DNA}40\ \left(1,\ 2\right)} - {C}_{\mathrm{DNA}60\ \left(1,\ 2\right)}\right){L}_{DNA}/{A}_{\mathrm{Eff}} $$

The effective area will be discussed in the following section: we can get the best values of fitting parameters if we fit the experimental data to Eqs. (10, 11, 14, and 15).

## Results and Discussion

### Effective Surface Area Between DNA Molecule and Metallic Electrodes

It is important to estimate the average magnitude of the effective area between the DNA molecule and the electrodes *A*_Eff_. This parameter has an essential role in the charge transfer mechanism because it connects two different domains: macro- and nano-area: DNA in nanoscale and metallic electrode in another greater scale. From theoretical point of view, if one considers DNA molecule as nanotube of radius 2 nm, this will lead to a theoretical area about 1.26 × 10^−17^ m^2^.

Thus, we tailored a sample holder, illustrated in Fig. 3a, in such a way that we measure, instantaneously, the electrical conductivity of DNA molecule and measure the electrical conductivity of a standard material (silicon nanowire) with well-known dimensions and values of dielectric permittivity of nanocomposites. Si nanowire has an electrical conductivity 3 × 10^−6^ Ω^−1^ cm^−1^, and dielectric constant 5ε_0_ given by the supplier (Sigma-Aldrich Chemicals Seelze GmbH, Frankfurt, Germany).

Once we have measured the exact electrical conduction and capacitance at certain length, we can estimate the effective area from the supplier-given conductivity and dielectric constant of the standard material. Our experimental data show that the effective area data is 2.1 × 10^−9^ m^2^.

Alternative technique to estimate the effective area is the following: we have started with the recently published data on the dielectric constant of DNA = 8ε_0_ at 1 MHz with ε_0_ as the space dielectric constant [41]. Then we measure the capacitance of DNA at 1 MHz for four different lengths at 40, 60, 80, and 100 nm. The values of capacitances corresponding to four different lengths of DNA molecule are 4.85 × 10^−12^ F, 3.22 × 10^−12^ F, 2.38 × 10^−12^ F, and 1.92 × 10^−12^ F, respectively. It is worth noting that at 1 MHz, we completely neglected the effect of the blocking conducer. At this high frequency, we considered that two parallel capacitances, *C*_DNA_ and *C*_ENV_, could adequately represent the measured capacitances. Taking parallel plate formula, for a certain length of DNA, *L*_DNA_ one will have the measured capacitance as *C*_MES_: *C*_MES_ = *C*_DNA_ + *C*_ENV_ = *ε*_DNA_*(A/*L*_DNA_) + *C*_ENV_. *A* is the effective area connecting the DNA molecule to the electrodes. One will have *ε*_DNA_ by simply subtracting two experimental capacitances as follows:

$$ {C}_{\mathrm{MES}40}-{C}_{\mathrm{MES}60}={C}_{\mathrm{DNA}40}+{C}_{\mathrm{DNA}60}={\varepsilon}_{\mathrm{DNA}}* A/40\mathrm{nm} - {\varepsilon}_{\mathrm{DNA}}*\mathrm{A}/60\mathrm{nm} $$

The effective area due to attachment of DNA with electrical contacts can be estimated, thus, as follows: *A* = (*C*_MES40_−*C*_MES60_)/[8*ε*_0_*(1/40 nm−1/60 nm)] = 2.72 × 10^−9^ m^2^. Repeating for the other lengths and taking a simple average, the effective area *A* = 2.74 × 10^−9^ m^2^. This value is not too far from the measured value against a standard nanowire: 2.21 × 10^−9^ m^2^.

### DC *I*-*V* Characteristics with Potential Fluctuations of the Extended Bands

**Fig. 4:**
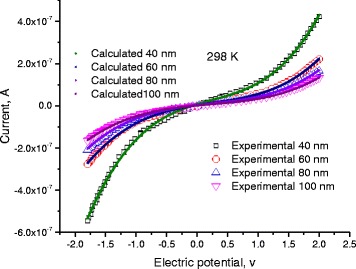
*I*-*V* characteristics at different lengths of DNA molecule: *L*_DNA_ 40, 60, 80, 100 nm

At room temperature, we measured *I–V* characteristics at different lengths of DNA molecule. Figure 4 shows the current behavior as a function of voltage for different lengths of DNA molecule: *L*_DNA_ 40, 60, 80, and 100 nm.

We fitted the experimental data (symbols) to Eqs. (5–7) where Tables 1, 2, 3, and 4 list the best fitting parameters. Continuous lines in Fig. 4 represent calculated data, and symbols stand for experimental data.

**Table 1:** Fitting parameters for AC-complex conductivity: electric conductivity and dielectric constant

Δ*E* (eV)	(H1)	(H2)	(H3)	(H4)	(H5)
	0.72	0.58	0.33	0.24 eV	0.05
ε’_DC_	3500	82	28	16	12
ε’_∞_	28.95	12.2	5.97	5.33	3.95

**Table 2:** Relaxation times of five energy levels corresponding to H1, H2, H3, H4, and H5; these times are used in fitting processes for AC-complex conductivity: electric conductivity and dielectric constant

*T* (*K*)	*τ* (H1)	*τ* (H2)	*τ* (H3)	*τ* (H4)	*τ* (H5)
288	0.648023032	0.004378409	0.000234262	2.43124E-05	1.12337E-06
293	0.395160894	0.002939474	0.000186743	2.06168E-05	1.08544E-06
298	0.245059221	2.01483E-03	1.54218E-04	0.00001758	0.00000105
303	0.154315643	0.001378192	0.000121361	1.50695E-05	1.01683E-06
308	0.09866699	0.000961259	9.88672E-05	1.29824E-05	9.85733E-07
313	0.063994035	0.000678219	8.1072E-05	1.12377E-05	9.56536E-07
318	0.042074608	0.000483796	6.68959E-05	9.7717E-06	9.29082E-07
323	0.028024575	0.000348736	5.5528E-05	8.53381E-06	9.0323E-07
328	0.018898986	0.000253902	4.63544E-05	7.48358E-06	8.78854E-07
333	0.012896627	0.000186627	3.89068E-05	6.58854E-06	8.55838E-07
338	0.008900695	0.000138432	3.28254E-05	5.82244E-06	8.34079E-07
343	0.006209649	0.000103582	2.78321E-05	5.164E-06	8.13484E-07

**Table 3:** DC electric conductivity of five energy levels corresponding to H1, H2, H3, H4, and H5; these times are used in fitting processes for AC-complex conductivity: electric conductivity and dielectric constant

*T* (*K*)	*σ*_DC_ (H1)	*σ*_DC_ (H2)	*σ*_DC_ (H3)	*σ*_DC_ (H4)	*σ*_DC_ (H5)
288	2.94907E-08	3.56306E-08	4.99457E-08	5.64029E-08	7.29078E-08
293	4.83617E-08	5.30726E-08	6.2655E-08	6.65131E-08	7.54558E-08
298	7.80028E-08	7.80028E-08	7.80028E-08	7.80028E-08	7.80028E-08
303	1.23842E-07	1.13196E-07	9.64104E-08	9.09975E-08	8.05475E-08
308	1.93689E-07	1.62293E-07	1.18345E-07	1.05627E-07	8.30885E-08
313	2.98633E-07	2.30023E-07	1.44322E-07	1.22026E-07	8.56248E-08
318	4.54211E-07	3.22463E-07	1.74906E-07	1.40333E-07	8.8155E-08
323	6.81929E-07	4.47348E-07	2.10713E-07	1.6069E-07	9.06783E-08
328	1.01121E-06	6.14436E-07	2.52414E-07	1.83241E-07	9.31935E-08
333	1.48185E-06	8.35929E-07	3.00732E-07	2.08134E-07	9.56999E-08
338	2.14712E-06	1.12695E-06	3.56448E-07	2.3552E-07	9.81965E-08
343	3.07761E-06	1.50612E-06	4.20397E-07	2.65551E-07	1.00683E-07

**Table 4:** Disorder energy of five energy levels corresponding to H1, H2, H3, H4, and H5; these times are used in fitting processes for AC-complex conductivity: electric conductivity and dielectric constant

(eV)	g*γ	g*γ	g*γ	g*γ	g*γ
*T*(*K*)	H1	H2	H3	H4	H5
288	0.206145132	0.196868601	0.185530619	0.181820007	0.164916106
293	0.202900677	0.193770147	0.182610609	0.17977	0.162726343
298	0.199478837	0.190502289	0.179530953	0.175940334	0.159583069
303	0.195892756	0.187077582	0.17630348	0.173560982	0.15710599
308	0.192156248	0.183509217	0.172940623	0.169481811	0.153724998
313	0.188283238	0.179810492	0.169454914	0.166818949	0.151003157
318	0.184287324	0.175994394	0.165858591	0.16254142	0.147429859
323	0.180181459	0.172073294	0.162163313	0.159640773	0.14450553
328	0.175977743	0.168058744	0.158379969	0.155212369	0.140782194
333	0.171687297	0.163961369	0.154518567	0.152114945	0.137693212
338	0.167320221	0.159790811	0.150588199	0.147576435	0.133856177
343	0.162885591	0.15555574	0.146597032	0.144316634	0.130634244

**Fig. 5:**
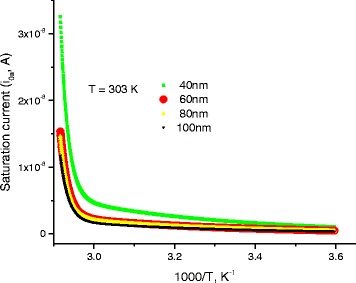
The saturation current estimated after the fitting processes of experimental data in Fig. 4 to Eqs. (5, 6, and 7)

**Fig. 6:**
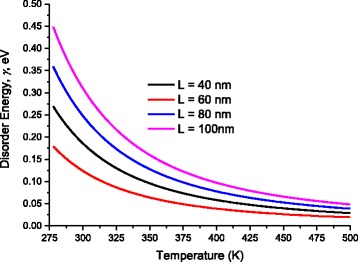
The disorder energy of DNA molecule for different lengths estimated after fitting of experimental data to Eqs. (5–11)

Scattering of charges on potential barriers in the extended bands increases when DNA-length increases and therefore, the mobility will be reduced leading to feeble values of current; this is in harmony with the fact that charges in long DNA (with long trajectory of charges) suffer more electrical resistance. Therefore, this leads to reduction of electric current and increases of electrical resistance. From the fitting processes, we remarked that the saturation current increases at higher temperatures (Fig. 5). In addition, Fig. 6 illustrates the reduction of the maximum WHs by temperature for different molecule lengths. We built this figure by looking, point by point, for the best value that fits well experimental electric current to Eqs. (5–7).

**Fig. 7:**
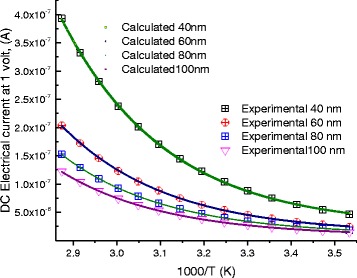
DC-current as a function of temperature for different lengths of DNA molecule. *Symbols* represent experimental data, and *continuous lines* are calculated data after Eqs. (5–7)

**Fig. 8:**
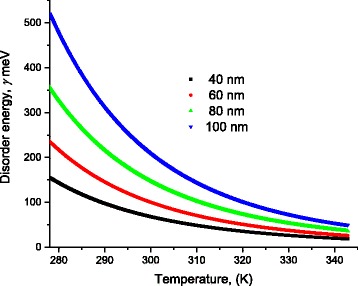
Variation of the disorder energy γ as a function of temperature for different DNA lengths

**Fig. 9:**
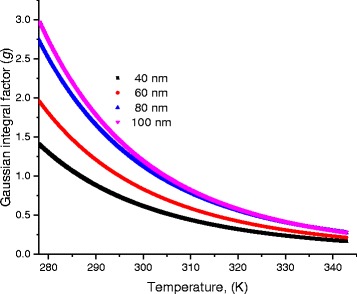
Dependence of the Gaussian factor (*g*) as a function of temperature for different DNA lengths

At 1 V, we reported the electric current as a function of temperature on Fig. 7; these curves are proportional to the electrical conductance *G*, Ω^−1^. The experimental data, in Fig. 7, are well fitted to Eqs. (5–7) with the fitting parameters shown in Figs. 8 and 9, and the parameters stated in Tables 1, 2, 3, and 4. After the previous model, more localized charges will be present with longer molecule, which means decrease in electrical current. Figure 7 shows clearly this behavior.

Figure 8 shows the values of the disorder energy γ that gives best fitting of experimental data to Eqs. (6 and 7).

As one can see from Fig. 8, the disorder energy increases with the molecule length while it decreases with temperature.

**Fig. 10:**
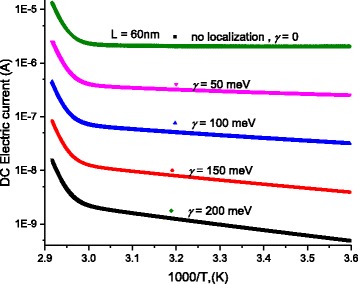
The DC-electric current, calculated from Eq. (6), as a function of temperature for different disorder energy

Figure 10 shows that disorder energy reduces the electric current passing through DNA molecule that confirms our abovementioned analysis about as the charge-scattering increases (due to increase of disorder), the mobility decreases and the electric current decreases.

### Thermal Effect on *I–V* Characteristics

**Fig. 11:**
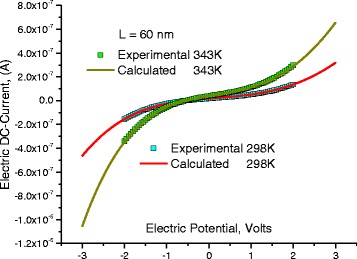
*I*-*V* characteristics for DNA molecule at different temperatures, *lines* represent calculated values after Eqs. (5–7) and *symbols* represent experimental values

Fixing the length of DNA molecule at 60 nm, Fig. 11 shows *I–V* characteristics at a fixed length 60 nm for different temperatures. The experimental data were well fitted to Eqs. (5–7) using the same parameters shown in Tables 1, 2, 3, and 4 and Fig. 11. The net increase of current with temperature is due to the shrinkage of potential hills with temperature. The reduction of the hills-depth allows more localized holes to arise over the most probable energy in the valence band and thus they become free and can conduct the electric power which increases, in turn, the electric current.

**Fig. 12:**
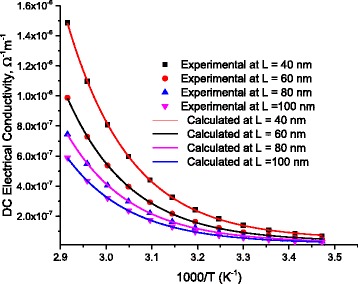
DC-electrical conductivity, calculated from Eq. (6-a), as a function of temperature for different DNA-lengths

Figure 12 illustrates the DC-conductivity as a function of temperature. Symbols represent experimental data while lines are calculated values after Eqs.6 and 6-a.

### AC Electrical Conductivity: Effect of Environmental Factors and Localization of Charges

**Fig. 13:**
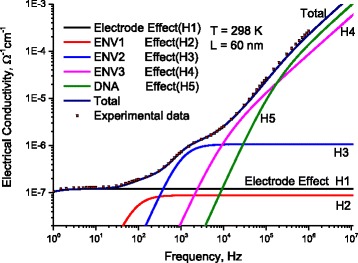
At 298 K, the measured AC-conductivity as a function of frequency for DNA molecule as a function of applied frequency, *lines* represent calculated values after Eq. (12) with the data in Figs. 8 and 9. *Symbols* represent experimental values. There are five humps marked as H1, H2, H3, H4, and H5

At room temperature with 60 nm DNA-length, we report AC-conductivity as a function of frequency (Fig. 13). In this figure, symbols represent the experimental ac-conductivity while continuous small dots (nearly lines with a frequency increment between the points 0.0137 Hz.) represent calculated values after Eq. (12). As seen in Fig. 13, one can notice the presence of five humps. By the abovementioned subtracting technique, we succeeded to divide *σ*_MES_ into clear five well-defined conductivities: *σ*_Electrodes_, *σ*_ENV1_, *σ*_ENV2_, *σ*_DNA1_, and *σ*_DNA2._ We will denote these five humps: H1, H2, H3, H4, and H5 in such a way that H1 lies at the lowest frequency, and H5 corresponds to the highest frequency. We noticed, also, that in the frequency range *f* < 1 Hz the conduction by bound charges and dipoles at the interface between DNA and metallic electrode is inferior to all the other conductivities. This happens from either environmental factors or DNA: σ_Electrodes_ < < *σ*_ENV1_, *σ*_ENV2_, *σ*_DNA1_, and *σ*_DNA2_; in the frequency range 10 < *f* < 100 Hz, σ_DNA1_, σ_DNA2_ ≈ σ_ENV1_, and *σ*_ENV2_; in the frequency range 200 < *f* < 10 kHz, σ_DNA1_, σ_DNA2_ > > *σ*_ENV1_, and *σ*_ENV2_; in the frequency range *f* > 10 kHz, σ_DNA1_, σ_DNA2_ > > *σ*_ENV1_, and *σ*_ENV2_.

Tables 1, 2, 3, and 4 shows the parameters used to estimate *σ*_MES_ (at 298 K); one notes satisfactory fitting processes between experimental and calculated values, for example:

*σ*_dcENV1_ = 5.1 × 10^−8^ Ω^−1^ m^−1^, *τ*_0ENV1_ = 7.89 × 10^−6^ s *τ*_0ENV1_ = 7.89 × 10^−6^ s *τ*_0ENV1_ = 7.89 × 10^−6^ s ε_dcENV1_ = 82.1 × 8.855 × 10^−12^ Fm^−1^, *ε*_∞ENV1_ = 4.2 × 8.855 × 10^−12^ Fm^−1^, γ_ENV1_ = 0 meV, and *g* = 1.87. The parameters used to estimate *σ*_DNA1_ are *σ*_dcDNA_ = 7.8 × 10^−8^ Ω^−1^ m^−1^, *τ*_0DNA_ = 3.9 × 10^−4^ s *ε*_dc DNA_ = 8.2 × 8.855 × 10^−12^ Fm^−1^, *ε*_∞DNA_ = 2.4 × 8.855 × 10^−12^ Fm^−1^, γ_DNA_ = 121.5 meV, *g* = 1.04, and *L*_0_ = 45 nm.

### AC Electrical Conductivity: Thermal Effect on Localized Charges

**Fig. 14:**
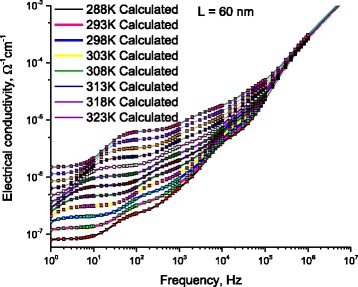
AC-conductivity as a function of frequency for DNA molecule as a function of frequency, for different temperatures. *Lines* represent calculated values after Eq. (12) with the data in Figs. 8 and 9. *Symbols* represent experimental values. *Lines* on figure illustrate the effect of temperature on four humps (H2, H3, H4, and H5). Hump H1 is independently represented in Fig. 15 because the scale, here, is not suitable to represent all H1 data

Fixing the length of DNA molecule at 60 nm, Fig. 14 illustrates the measured AC-conductivity as a function of frequency for DNA molecule, for different temperatures. Lines represent calculated values after Eq. (12) with the data in Figs. 8 and 9. Symbols represent experimental values. Figure 14 illustrates the thermal-effect on four humps (H2, H3, H4, and H5). Noting that the temperature effect on the hump H1 is not well manifested in Fig. 13; therefore, we repeated the experiment in the frequency range 0.05Hz < *f* < 10 Hz. The frequency-step was 0.05 Hz (note that the Solartrone 1260A has the validity to detect 10^−4^ Hz variations, which was not possible at 2011; the time of our previous study in reference [7]). These fine measurements allowed us to examine what is happing in the very low frequency range. Figure 14 shows the variations of the electrical conductivity as a function of frequency at very low frequencies, for different temperatures. One notes the smooth variations of H1 with both temperature and frequency. This behavior is, in fact, important because it leads to obtain the value of charges-relaxation-time as a function of temperature as we will see in the next section.

**Fig. 15:**
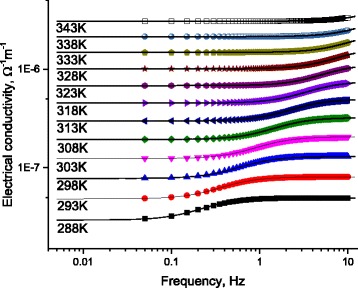
Low frequency dependence of the AC-conductivity for DNA molecule and for different temperatures (H1). *Lines* represent calculated values after Eq. (12) with the data in Figs. 8 and 9. *Symbols* represent experimental values. One can see the effect of temperature on H1

The measurements in Fig. 15 show that the charges bound at the DNA/electrode surface relax depending on temperature. The temperature dependence of electric losses leads directly to get the relaxation time as a function of temperature.

### Effect of Relaxation of Charges on the AC-Electrical Conductivity

When applying AC-electric field on electric charges found through DNA or around it, these charges will respond with certain relaxation time depending on their energy. They will transfer electric current if their own relaxation time fit the inverse of the angular frequency. This is well seen when plotting the electric losses ε” as a function of frequency and temperature. The relaxation time of charges corresponds the maximum of ε”−ω curve at certain temperature. This relaxation time is an intrinsic property of charges whether they are on the molecules or around it and whether they are dipoles, ions, or free charges. One can calculate the relaxation time after the measured values of electrical conductivity data σ_MES_ from the following relation:

16 $$ \varepsilon "=\frac{\left({\sigma}_{\mathrm{MES}}-{\sigma}_{\mathrm{DC}}\right)}{\omega}\Big) $$

Where *ω* is the radiant frequency ω = 2π*f*.

**Fig. 16:**
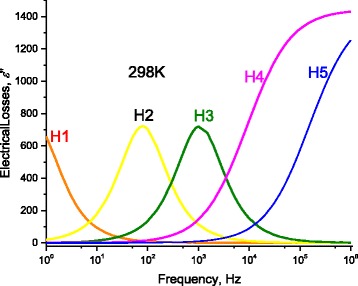
At 298 K, the electric losses ε” as a function of frequency for DNA molecule. The relaxation times of H2, H3, H4, and H5 correspond to the maximum frequencies as *arrows* point out. One can better see the effect of frequency on H1 at lower frequencies in next figure

**Fig. 17:**
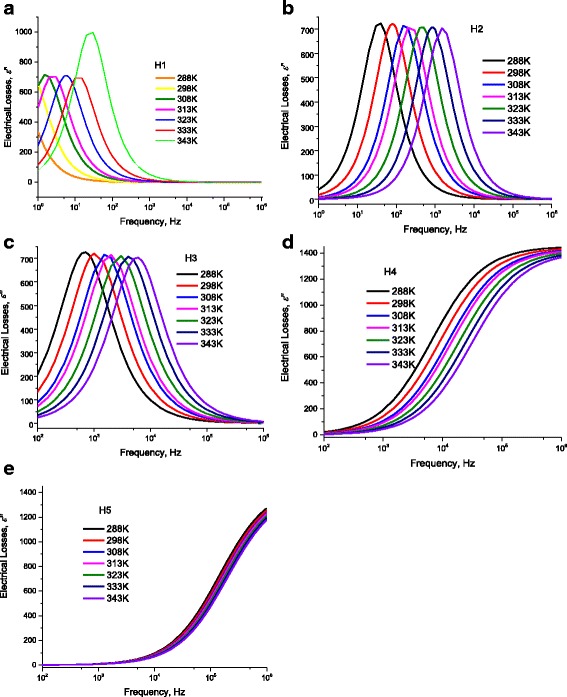
**a** Electric losses as a function of frequency calculated after AC-conductivity through DNA molecule for different temperature humps (H1). *Lines* represent calculated values after Eq. (12) with the data in Figs. 8 and 9. **b** Electric losses as a function of frequency calculated after AC-conductivity through DNA molecule for different temperature humps (H2). *Lines* represent calculated values after Eq. (12) with the data in Figs. 8 and 9. **c** Electric losses as a function of frequency calculated after AC-conductivity through DNA molecule for different temperature humps (H3). *Lines* represent calculated values after Eq. (12) with the data in Figs. 8 and 9. **d** Electric losses as a function of frequency calculated after AC-conductivity through DNA molecule for different temperature humps (H4). *Lines* represent calculated values after Eq. (2) with the data in Figs. 8 and 9. **e** Electric losses as a function of frequency calculated after AC-conductivity through DNA molecule for different temperature humps (H5). *Lines* represent calculated values after Eq. (12) with the data in Figs. 8 and 9

At certain length of DNA *L* = 60 nm, Fig. 16 illustrates the electric losses ε” through DNA molecule as a function of the applied frequency at 298 K. One can see from this figure that the previously observed humps on the conductivity curve correspond to well definite maximum at certain temperature (Figs. 16 and 17a –e). We observe three maxima relating to H1, H2, and H3. However, because of the abovementioned shortage of scale, we reported the maximum of H1 on Fig. 15. We believe that dipoles are bounded in the thin area between the DNA surface and metallic electrodes which explains why ε’ has strong values. H1 is attributed to the presence of bound charges at the metallic-electrode/DNA surface. The Gaussian distribution of relaxation times of these charges is very narrow and they acquire a maximum. Similarly, the weakly attached charges to DNA have very narrow and they acquire two maxima. On the contradictory, H4 and H5 are attributed to the intrinsic charges on DNA which are localized in potential wells/hills. The localization at different energy states allows for these charges to have wide Gaussian distribution of relaxation times which do not give any maxima. This is also seen in Cole and Cole curves where H1, H2, and H3 have semicircular behavior while H4 and H5 have not due to the wide distribution of relaxation times on the intrinsic DNA charges. Similarly, the high value of ε” reflects the presence of dipoles with “relative high density” in very thin spacing. However, one needs additional C-V experimental data to confirm that these dipoles have high density and to get the exact density of these surface dipoles, which will be the subject for another study. As one can see from the inset of Fig. 15, at 298 K, the maximum of the electric losses curve lies at 0.649 Hz. Similarly, one obtains maximum frequencies for H2, H3, H4, and H5 as 79.617, 1061.176, 9057.774, and 151653 Hz, respectively, that corresponds to relaxation times for H1, H2, H3, and H4 as 2.00 × 10^−3^, 1.510 × 10^−4^, 1760 × 10^−5^, and 1.051 × 10^−6^ s, respectively. The magnitudes of maxima depend on (and are related with) the charges density. As seen in Fig. 15 and in Tables 1, 2, 3, and 4, when we fitted the experimental data, at 298 K, to Eqs. (12) and (13), we have used disorder energies corresponding to H1, H2, H3, H4, and H5 as γ = γ_Electrodes_ = zero, γ_ENV1_ = zero, γ_ENV2_ = zero, γ_DNA1_ = 121.5 meV, *g* = 1.04, and γ_DNA2_ = 56.01 meV, *g* = 1.37. These fitting parameters give an initial conclusion that there are no disorder at H1, H2, and H3. One can confirm the abovementioned conclusions: dipoles at the interface between metallic electrode and DNA are the reason of H1. The other two humps are attributed to the presence of free charges, weakly bound charges (H_2_ bonds or Van der Waals ones) and ions surrounded to the molecule. The last two humps H4 and H5 are attributed to the charge conduction through a very complicated and disordered system: DNA. This is attributed to the fact that some charges are localized in potential hills through DNA molecule, and some other charges have sufficient energies to be free. We will give more confirmation for these conclusions in the following sections using another experimental data: for example, the temperature dependence of these relaxation times gives interesting confirmation. Figure 16a –d and f shows the temperature dependence of ε”−ω curves for H1, H2, H3, H2, H4, and H5. The maxima of these curves lead directly to the relaxation times corresponding to each hump. We calculated the relaxation time from the critical frequency *f*_c_ corresponds to the maximum of ε”−ω curve; for example, for H1, one can see the maximum at = 0.649 Hz which gives directly the relaxation time τ_H1_ = 1/(2* π * *f*_c_) 1/(2* π *0.649) = 0.24 s.

**Fig. 18:**
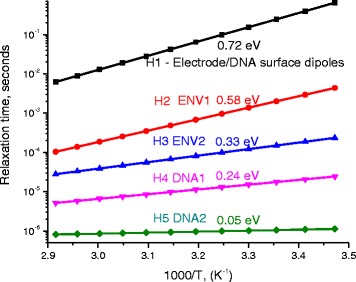
Relaxation time of electric charges on H1, H2, H3, H4, and H5 as a function of 1000/T (*K* ^−1^). The slopes of semilog lines are 0.72, 0.58, 0.33, 0.24, and 0.05 eV for charges on H1, H2, H3, H4, and H5, respectively

Figure 18 shows the values of relaxation times estimated after the experimental values of σ_MES_ and calculated from Eq. (16). Lines in the figure represent calculated values, and symbols are for experimental ones.

From Fig. 18, one notes the presence of five lines on the semilogarithmic scale indicating the presence of five thermally activated processes. We believe that these five processes are correlated with H1, H2, H3, H4, and H5. Therefore, we consider that the measured electrical conductivity contains five factors: the first factor (H1) appears at low frequency (about 0.64 Hz) with relaxation time 0.24 s. This effect is due to the relaxation of charges and dipoles at the interface between metallic electrode and DNA molecule. The thermal activation process show that this effect is thermally activated with an activation energy = 0.72 eV (Fig. 18). The intrinsic relaxation time of these charges and dipoles is *τ*_Electrode_ = 0.24 s. Resonance occurs when applying an external frequency *ε*_Electrodes’_ = 1/(2πτ_Electrode_). The second factor (H2) appears at higher frequency (79.6 Hz) with relaxation time 0.002 s. This effect is due to the relaxation of free charges and ions that present surrounding the DNA molecule. The thermal activation process shows that this effect is thermally activated with an activation energy = 0.58 eV. The intrinsic relaxation time of these charges and dipoles is *τ*_Electrode_ = 0.002 s. Resonance occurs when applying an external frequency *f*_Electrodes_ = 1/(2πτ_Electrode_) = 79.6 Hz. Therefore, this effect is attributed to the environmental factors; we denote their relaxation time and their conductivity as *τ*_ENV1_ and σ_ENV1_. The third factor is environmental factor ENV2 (H3), which is found at 1061.7 Hz with relaxation time 0.00015 s. We attribute his factor to the electrical conduction by free charges and ions that present surrounding the DNA molecule. The thermal activation process (Fig. 18) show that this effect is thermally activated with an activation energy = 0.33 eV.

The fourth and fifth factors (H4 and H5) appear at relatively higher frequencies (9057.97 Hz and 1.517 × 10^5^ Hz) with relaxation times 1.76 × 10^−5^ s and 1.05 × 10^−6^ s, respectively. We believe that the charges on DNA molecule are situated on a trapping level (TL) within the energy gap band at 0.24 and 0.05 eV. Therefore, the electric conduction due to H4 and H5 is attributed to the relaxation of charges that present on the DNA molecule. From Fig. 18, the thermal activation process shows that these two effects are thermally activated with activation energies = 0.24 and 0.05 eV, respectively. The intrinsic relaxation time of these charges and dipoles is *τ*_DNA1_ = 1.76 × 10^−5^ s. Resonance occurs when applying an external frequency *f*_DNA1_ = 1/(2πτ_DNA1_) = 9057.97 Hz. Therefore, this effect is attributed to the conduction by the molecule itself.

Therefore, DNA molecule is a wide-band semiconductor with two trapping levels at 0.05 and 0.24 eV.

### Dielectric Constant: Effect of Environmental Factors and Localization of Charges

Another experimental proof of the abovementioned analysis is measured values of dielectric constant.

**Fig. 19:**
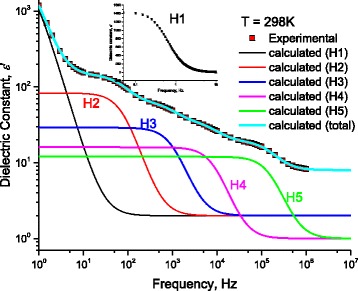
The frequency dependence of measured dielectric constant ε‘at 298 K. One notices the presence of H1, H2, H3, H4, and H5. H1 is presented in the *inset* of the figure

Figure 19 shows the frequency dependence of measured dielectric constant at 298 K. One notices the presence of H1, H2, H3, H4, and H5. Here, H1 is clearly manifested, and there is no need to repeat the measurement in below frequencies as we have done in the conductivity measurements. The measured dielectric constant at 298 K is well fitted to Eq. (11) with the same relaxation times previously obtained from ε”−*f* curves (Fig. 17a –e).

### Dielectric Constant: Effect of Environmental Charges on Localization of DNA Charges

**Fig. 20:**
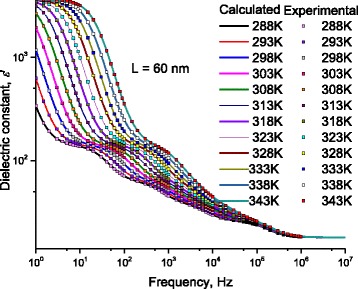
The frequency dependence of measured dielectric constant ε‘at different temperatures. One notices the presence of H1, H2, H3, H4, and H5

At DNA-length 60 nm, Figure 19 illustrates the measured dielectric constant as a function of frequency for DNA molecule at 298 K. Lines represent calculated values after Eq. (11) with the data in Figs. 8 and 9. Symbols represent experimental values. One can notice, from Figs. 19 and 20, the huge value of dielectric constant due to the participation of dipoles at the electrode interface: ε‘ = 1434.85ε_0_; where ε_0_ is the dielectric constant of vacuum. This high value drastically affects the experimental measurements. We believe that this high value of ε’is one important reason to get contradictory published data. Each author has his specific electrode resistance and specific environmental factors. This leads to several possibilities: first, if the electrode resistance is weak and specific, environmental factors are high conductors; this will give metallic electrical conductivity. Here, the environmental ions and charges superimpose the charges on DNA. Second possibility is if the electrode resistance is high and specific, environmental factors are poor conductors; this will give insulator electrical conductivity. Here, the electrode resistance superimposes both the environmental ions and charges the charges on DNA. Third possibility, which is less simple, is that if the electrode resistance is sufficiently low and specific, environmental factors are poor conductors; this will give semiconducting electrical conductivity. Here, the charges on DNA superimpose both electrode resistance and environmental ions. Therefore, the electrical metallic contacts should be carefully done. We think that it is a substantial point to ensure the performance of metallic contacts before any measurements. For this reason, we have subjected our electrical contacts to UV radiation before and after connecting DNA molecule to the metallic electrodes. The inset in Fig. 3 shows that there were good electrical contacts.

### Dielectric Constant: Effect of Temperature on Localization of Charges

**Fig. 21:**
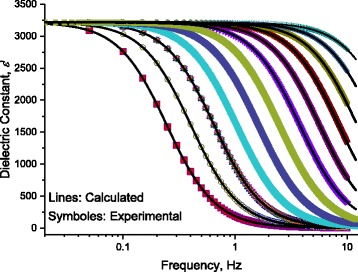
At low frequency range, for H1: dielectric constant dielectric constant, ε’ measured as a function of frequency at different temperatures. *Symbols* represent experimental values, and *lines* represent calculated values after Eq. (11); for the temperatures 288, 293, 298, 303, 303, 308, 313, 318, 323, 328, 333, 338, and 343 K, where *red rectangles* represent values of ε’ at 288 K, and *yellow circles* represent value of ε’ at 293 K, respectively

Fixing the length of DNA molecule at 60 nm, Fig. 20 illustrates the measured dielectric constant, ε‘as a function of frequency for DNA molecule, for different temperatures. Lines represent calculated values after Eq. (12) with the data in Figs. 8 and 9. Symbols represent experimental values. The effect of temperature on five humps (H1, H2, H3, H4, and H5) is well manifested. Here, the temperature effect on the hump H1 is well manifested; therefore, we are not in need of more investigations at lower frequencies. One notes the smooth variations of H1 with temperature. These smooth variations are well illustrated in Fig. 21 where dielectric constant is traced as a function of frequency for different temperatures. It is worth noting that the values of static dielectric constant shown in this figure and stated in Tables 1, 2, 3, and 4 affect drastically the electrical conduction through DNA molecule. When a scientific researcher measures the conductivity through DNA and does not take account for these dipoles lying between the electrode and DNA, he will get deviated measurements and conclusions.

The behavior shown in Figs. 20 and 21 is in complete harmony with the abovementioned σ_MES_ data. In order to confirm the abovementioned analysis, we will present our experimental data in Cole-Cole form, as we will see in the next section.

### Cole-Cole Presentation

Another mathematical way to support the abovementioned analysis is to present the experimental data in Cole-Cole curves. Cole first introduced the idea in 1940 [42] to fit experimental data obtained by bioimpedance measurements. The model considers four parameters: *R*_DC_, *R*_∞_, *τ*, and *α* with their common definition. *τ* is the inverse of the natural characteristic frequency *f*_c_, and *α* correlates with the departure of a model of a simple RC circuit model. One writes down Cole equation as:

17 $$ R={R}_{\mathrm{DC}}\frac{\varDelta {\varepsilon}_0}{\left[1+{\left( j\omega \tau \right)}^{\alpha}\right]}; j=\sqrt{-1} $$

Cole model [32] considers plotting resistance (or electric losses) vs. reactance (or dielectric constant). For *α* = 1, the Cole equation is linear with respect to frequency, and the generated impedance values represent a complete semicircle if plotted in the impedance plane. For *α* ≠ 1, one obtains a suppressed semicircle.

Moreover, in reference [43], the author related between the Cole parameter *α* and the abovementioned disorder energy *γ*. In that work [43], the author showed that the ideal relaxation that obeys (completely) Debye relaxation with *α* = 1 corresponds to the most ordered system which contains zero disorder defects. This means that *γ* → 0 when *α* → 1 and *γ* → maximum when *α* → 0.

**Fig. 22:**
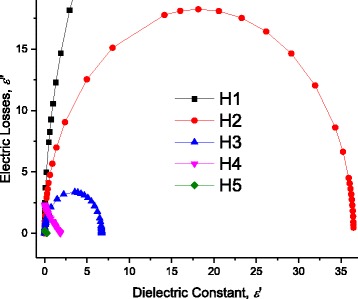
Cole-Cole graph: dependence of electric losses as a function of dielectric constant at temperature = 298 K. One notices that H1, H2, H3, H4, and H5 have different behaviors

**Fig. 23:**
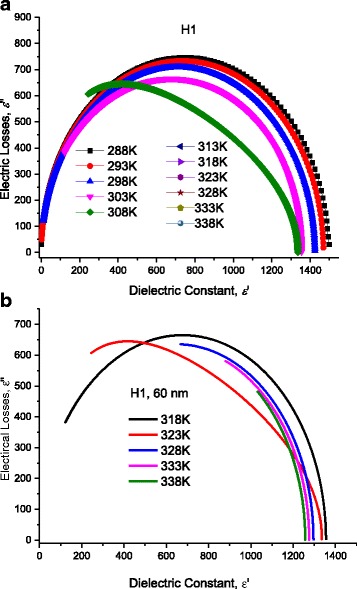
Cole-Cole graph: dependence of electric losses as a function of dielectric constant for the charges at the interface between electrode and DNA (H1) at different temperatures. One notices that presence of nearly complete semicircle

In the present work, we illustrate the electric losses as a function of the dielectric constant (Cole-Cole curves) at 298 K in Fig. 22. One notes that H1 is out of scale; therefore, we replotted it, for different temperatures, indently in Fig. 23. We performed more studies for H1 in Fig. 23 in order to study the behavior of charges (dipoles) at low frequencies. From Figs. 22 and 23, one confirms the presence of complete semicircles for H1, H2, and H3, which reflects the nature of homogeneity in H1, H2, and H3. For H1 and H2, we attribute their homogeneity to the ability of free charges and free ions to move around the molecule and through its environments. Therefore, the semicircles of H2 and H3 give another confirmation that the measured AC-conductivity includes measurements due to charges around DNA molecule. In addition, charges at the interface between metallic electrode and DNA molecule are distributed in homogeneous manner. We believe that the majority of charges at this interface form dipoles with strictly ordered distribution due to the limitations of space. Therefore, one finds nearly perfect semicircles, which reflects the homogeneities of the charge-distribution on H1. However, at relatively high temperature 343 K, there are some deviations from the semicircle behavior (Fig. 23). We attribute this behavior to strong diffusion of charges coming from the “hot-molecule” towards the interface.

The semicircles behavior of H1 is seen once more when one plots *ε*’ as a function of *ε*” for different temperatures. Complete semicircles reflect the absence of disorder even in relatively low temperatures (288); except at 343 K where the molecule starts to change its phase. This shows that charges located at the boundary between DNA and metallic electrode are homogeneously distributed. As we have stated above, the nature of charges in the area between DNA and metallic electrode is strongly bounded dipoles. We believe that the majority of published data have these strongly bounded dipoles at the end of DNA molecules except for some rare work such that of Kasimov et al. [6] and that of Porath et al. [44], which gives another confirmation that the measured AC-conductivity includes measurements due to charges around DNA molecule.

**Fig. 24:**
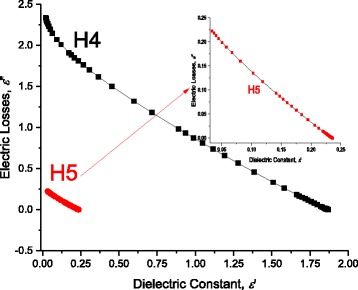
Cole-Cole curves for the charges at DNA (H4 and H5) at different 298 K. One notices that there is complete departure from the “semi-circle behavior”

On the contradictory, Fig. 24 shows Cole-Cole curves for charges on H4 and H5. As seen, there is linear dependence without any tendency for a semicircle response. This is due to the strong disordered state of molecule-structure. The linearity between *ε*’ and *ε*” reflects the presence of strong disordered structures which results in localization of charges in potential wells (hills).

## Conclusions

We have characterized λ-double helix DNA using AC-electric field using comprehensive and firm control experiments to ensure the validity of contacts between metallic electrode and molecule. In the following, we will give brief statements of the principal points in the present work:

- First, the environmental charges, ions, and dipoles drastically affect the electrical conduction mechanism through DNA. From electrical point of view, DNA molecule behaves as a wide-energy-gap semiconductor
- Second, due to the abovementioned eight disorder factors, in particular the complicated structure of DNA molecule, the disorder effect leads to creation of potential wells and hills in the extended band where charges are localized. Charges are present in localized energy states (LESs). Localization of charges in the extended bands controls the main mechanism of charge transfer through the molecule. Up to the present study, there are three main types of charges that contribute to the charge transfer through DNA molecule: (i) bound dipoles and bound charges at the interface between metallic electrode and DNA, (ii) environmental charges and ions around the molecule, (iii) charges (either free or localized) on the molecule itself. These three factors result in five humps on the electrical conductivity as a function of frequency (and the same for the dielectric constant), as we will see in the experimental results section. Good estimation of the principal role of surrounding charges, which are present around the molecule and at the electrode surface, is a substantial point for studying electrical conduction through DNA molecule, and it should be accompanied by ensuring effective metallic electrodes. Our results could restimulate interest in DNA-based nanodevices in particular in the medical, medical-engineering and future computers, intelligent nano devices, biosensing, DNA-repair, and different other applications.

## Abbreviations

*C*_∞_: Measured capacitance at very high frequencies
CB: Conduction band
*C*_dc_: Measured capacitance at very low frequencies
CT: Charge transfer
DNA: Deoxyribonucleic acid
*E*_AC_: AC-field
*E*_C_: Energy state of the conduction band
*E*_C0_: Most probable value in the conduction band within the Gaussian distribution
EFs: Environmental factors
Eg: Forbidden gap
*E*_V_: Energy state of the valence band
*E*_V0_: Most probable value in the valence band within the Gaussian distribution
*f*: Frequency
*g*: Gaussian-integral limits
*G*_∞_: Measured capacitance at very high frequencies
*G*_dc_: Measured conductance at very low frequencies
gγ: Depth of the potential well
H: Trapping hole-level (H1, H2, H3, H4, H5)
HOMO: Highest occupied molecular orbital
*i*_drift_: Drift current through a diode
*i*_gen_: Generation current through a diode
*i*_sat_: Saturation current through a diode
*L*: Length of molecule
LESs: Localized energy-states
LUMO: Lowest unoccupied molecular orbital
P(E_C_): Gaussian probability to find certain localized energy state within the conduction band
P(E_V_): Gaussian probability to find certain localized energy state within the valiancy band
*R*_DNA_*C*_DNA_: Parallel electronic circuit represents the electrical effect of charges through DNA molecule
*R*_EVN_*C*_ENV_: Parallel electronic circuit represents the electrical effect of the environmental charges, ions, and dipoles
*T*: Temperature
*u*: Reduced energy *u* = gγ/kT
VB: Valence band
*α*: Constant of dimensions nm.eV^−1^
*ε*’_DNA_: Dielectric constant due to DNA
*ϕ*: Potential barrier, eV
*γ*: Disorder energy
*σ*_DNA_: Electrical conductivity due to DNA
*σ*_ENV_: Electrical conductivity due to EFs
*σ*_MES_: Measured conductivity
*τ*: Relaxation time of electric charges
*τ*_0_: Relaxtion time of charge without disorder (without localization)
